# Exploring Lectin Bioactivity and Total Phenolic Compounds in Kiwifruit (*Actinidia deliciosa* var. Hayward)

**DOI:** 10.3390/nu16193292

**Published:** 2024-09-28

**Authors:** Raquel Rodrigues, Maria Eduardo Figueira, Rosa Direito, Andreia Bento-Silva, Ricardo Boavida Ferreira, Ana Cristina Ribeiro

**Affiliations:** 1 Faculdade de Farmácia, Universidade de Lisboa, Av. Prof. Gama Pinto, 1649-003 Lisboa, Portugal; raquelines@edu.ulisboa.pt (R.R.); abentosilva@ff.ulisboa.pt (A.B.-S.); acribeiro@ff.ulisboa.pt (A.C.R.); 2 Linking Landscape, Environment, Agriculture and Food (LEAF), Instituto Superior de Agronomia, Universidade de Lisboa, 1349-017 Lisboa, Portugal; rbferreira@isa.utl.pt; 3 Laboratory of Systems Integration Pharmacology, Clinical and Regulatory Science, Research Institute for Medicines, Universidade de Lisboa (iMed.ULisboa), Av. Prof. Gama Pinto, 1649-003 Lisbon, Portugal; rdireito@ff.ulisboa.pt

**Keywords:** kiwifruit, *Actinidia deliciosa* var. Hayward, bioactivities, phenolic compounds, lectins

## Abstract

Background: The consumption of kiwifruit (*Actinidia deliciosa* var. Hayward) is recognized for its health benefits due to its high vitamin C content and bioactive secondary metabolites, such as phenolic compounds with antioxidant properties. These compounds may help prevent chronic noncommunicable diseases, currently the leading cause of death. Additionally, plants and fruits contain proteins like lectins, which contribute to plant defense and may also have health-promoting effects, including antitumor and hypoglycemic activities. Objectives: The objective of this work was to evaluate and identify the phenolic compounds in this variety of kiwifruit, as well as to investigate the lectin activity and the potential dietary benefits of this combination. Methods: This study quantified and identified total phenolic compounds and flavonoids in a kiwifruit extract using HPLC-DAD-MS/MS, and assessed their antioxidant activity through the DPPH method. Results: Novel lectin activity was also investigated, with polypeptide characterization and glycoprotein profiling performed. The affinity of lectins for glycans was evaluated using a hemagglutination inhibition assay. Results indicated that kiwifruit lectins bind to glycoreceptors on tumor cell membranes, with a specific affinity for sialic acid, an important glycan in tumor-associated glycomic aberrations. Conclusions: These findings suggest that the bioactive components of kiwifruit may offer multiple health benefits through a synergistic effect.

## 1. Introduction

Kiwifruit (*Actinidia deliciosa* var. Hayward) originated from China and was exported to Europe in the 18th–19th centuries [1,2], but the cultivar Hayward was developed in New Zealand in the 1920s [3]. Later, it began to be produced in several countries due to its nutritional richness, appealing flavor, and associated health benefits.

Kiwifruit is a source of vitamin C, potassium, carotenoids, folate, and dietary fiber along with secondary metabolites and phenolic compounds. These compounds have demonstrated various bioactivities that provide health benefits [2,4], primarily due to their antioxidant and anti-inflammatory properties [4,5,6], which can help prevent noncommunicable diseases (NCDs) such as diabetes, cardiovascular disease, and cancer, the leading cause of death in developed countries [6]. Consequently, kiwifruit consumption has increased exponentially in recent decades.

Kiwifruit has also been recognized for its benefits to digestive, immune, and metabolic health, with digestive health being the most studied due to its ability to promote abdominal comfort [2]. Kiwifruit exhibits antioxidative, antiproliferative, anti-inflammatory, antimicrobial, antihypertensive, antihypercholesterolemic, neuroprotective, and antiobese properties and promotes gut health [4]. Several studies have explored the potential relationship between the bioactive components of kiwifruit and anticancer activity [7]. Findings suggest that kiwifruit supplementation is linked to anticancer effects, particularly due to ascorbic acid’s role in preventing cancer by balancing antioxidants and reactive oxygen species (ROS) [8].

There are differences between species and cultivars of kiwifruit, and therefore, different authors report varying values depending on the varieties used and the differing climatic conditions and cultivation terrains in which the fruits are grown [4,9,10,11,12].

Several publications indicate that the consumption of fruit and vegetables rich in phenolic compounds is associated with health benefits, including protection against certain cancers and the prevention of degenerative diseases, largely due to their antioxidant properties [5]. Antioxidants prevent free radical formation, remove radicals before damage occurs, repair oxidative damage, eliminate damaged molecules, and prevent mutations. They also inhibit cancer progression (metastasis), migration, and invasion and promote apoptosis [13,14].

Since kiwifruit has antioxidant and anti-inflammatory properties, its consumption is believed to have beneficial effects on NCDs as both oxidative stress and inflammation are known risk factors for these diseases. For this reason, the bioactivities of kiwifruit have been characterized based on its phenolic compound content and its antioxidant and anti-inflammatory properties. These properties may help combat factors associated with NCDs although clinically significant effects of kiwifruit on the prevention and/or treatment of major chronic diseases have yet to be confirmed. The presence of lectins in plants is already well established. In vitro and in vivo studies document their antitumor activity in various tumor cells [15,16], demonstrating inhibition of cancer cell proliferation, migration, and metastasis and inducing cell death through different mechanisms: apoptosis, necrosis, autophagy, and inhibition of angiogenesis [17,18,19].

The characterization of kiwifruit’s lectin bioactivities and phenolic compound content was based on lectin’s ability to bind glycoreceptors on tumor cell lines, indicating potential antitumor activities. It is important to note that lectins have never been previously found or described in kiwifruit. For the phenolic compounds of kiwifruit, their antioxidant properties result in anti-inflammatory effects, which are known to combat factors associated with NCDs. Lectins may also contribute to this effort, leading to potential improvements in public health. NCDs are considered one of the top 10 major health threats by the World Health Organization, responsible for the majority of deaths worldwide, with a significant portion being premature. Thus, research on foods that, through their bioactive compounds, may help address this global public health issue is critically important. The objective of this study is to examine the bioactivities of kiwifruit, focusing on its lectin and phenolic compound content, and exploring the potential contribution of lectins—so far not evidenced—when combined with other compounds in kiwifruit during consumption. Further research may reveal the possibility of therapeutic applications for this consortium of bioactivities.

## 2. Materials and Methods

### 2.1. Materials, Solvents and Reagents

Rabbit blood was purchased from Probiológica (Belas, Portugal), storage at 4 °C until to be used for hemagglutination assays. Human cells were all purchased from Sigma-Aldrich (St. Louis, MO, USA): HT-29 cells from colon adenocarcinoma (*Homo sapiens sapiens*), cell line ECACC No. 91072201, and MIA PaCa-2 Cells from pancreatic carcinoma (*Homo sapiens sapiens*), cell line ATCC No. CRL-1420 and were storage and preserved at −80 °C.

Absolute ethanol was sourced from Fisher Scientific (Loughborough, UK), distilled H_2_O was obtained from a Desminwater purification system (Lisbon, Portugal), and Whatman No. 41 filter paper was also used Folin–Ciocalteu reagent was obtained from Merck (Darmstadt, Germany), sodium carbonate from VWR Pro-lab (Leuven, Belgium), and gallic acid from Sigma-Aldrich (St. Louis, MO, USA). Epicatechin (98%) was obtained from Sigma-Aldrich (St. Louis, MO, USA), sodium nitrite (98%) from Panreac (Barcelona, Spain), aluminum chloride (99%) from Chem-Lab (Zedelgem, Belgium), and sodium hydroxide (1 M) from Fisher Scientific (Loughborough, UK). Formic acid ≥ 95% was obtained from Sigma-Aldrich (St. Louis, MO, USA), acetonitrile HPLC Plus Gradient grade was acquired from Carlo Erba (Val de Reuil, France), and water was purified by a Milli-Q water purification system from Millipore (Burlington, MA, USA). Trolox and 2,2-diphenyl-1-picrylhydrazyl (DPPH) from Sigma-Aldrich (St. Louis, MO, USA). Polyvinylpolypyrrolidone (PVPP) was acquired from Sigma-Aldrich (St. Louis, MO, USA), protease inhibitor cocktail (Complete, mini, EDTA-free) from Roche (Basel, Switzerland), and Tris and sodium citrate from Merck (Darmstadt, Germany). Acrylamide, bis-acrylamide, SDS, and 2-Mercaptoethanol were obtained from Sigma-Aldrich (St. Louis, MO, USA), glycine and ultrapure Tris from PanReac Applichem (Barcelona, Spain), glycerol from Merck (Darmstadt, Germany), m-Cresol purple from Sigma-Aldrich (St. Louis, MO, USA), Ponceau S from Schleicher & Schuell (Dassel, Germany) and BSA from Sigma-Aldrich (St. Louis, MO, USA), silver nitrate from Sigma-Aldrich (St. Louis, MO, USA), IEF-IPG strips (11 cm, pH 3–10 gradient) from Bio-Rad (Hercules, CA, USA), and urea from Merck (Darmstadt, Germany). Dithiothreitol, iodoacetamide, agarose, concanavalin A-lectin, and peroxidase were purchased from Sigma-Aldrich (St. Louis, MO, USA). The Fetuin separopore 4B column was acquired from BioWorld (Dublin, OH, USA) and ethanolamine from Merck (Darmstadt, Germany). Culture media: Roswell Park Memorial Institute (RPMI) and Dulbecco’s modified Eagle’s medium (DMEM); fetal calf serum, L-glutamine (200 mM), Penicillin G sodium salt, Streptomycin, Trypsin, and HEPES. Sucrose and EDTA were acquired from Merck (Darmstadt, Germany). NaCl from Biochem Chemopharma (Entrains-sur-Nohain, France); trypsin from porcine pancreas was acquired from Sigma-Aldrich (St. Louis, MO, USA). CaCl_2_ and MgCl_2_ from Merck (Darmstadt, Germany). For hemagglutination inhibition, a total of 17 sugars were assayed, all of which were purchased from Sigma-Aldrich (St. Louis, MO, USA): (1) d-glucose, (2) d-glucosamine, (3) *N*-acetyl-d-glucosamine, (4) d-galactose, (5) d-galactosamine, (6) *N*-acetyl-d-galactosamine, (7) lactose, (8) *D*-mannose, (9) raffinose, (10) l-fucose (0.3 M), (11) melezitose, (12) α-methyl-d-glucopyranoside, (13) α-methyl-d-mannopyranoside, (14) sucrose, (15) maltose, (16) sialic acid, and (17) l(−)-fucose (0.3 M).

### 2.2. Methods

#### 2.2.1. Extraction of Phenolic Compounds

*Actinidia deliciosa* var. Hayward (originally from Chile but grown in Portugal) was sourced from supermarkets as individual units, each with a certificate of variety and country of origin, and stored at −80 °C for later analysis.

Kiwifruit was peeled and blended, and a 90% ethanol (*v*/*v*) solution was added. The mixture was subsequently subjected to ultrasound for 20 min in the dark. Centrifugation was performed for 5 min, and then the supernatant was filtered through gauze. It was stored at −18 °C for later HPLC analysis.

#### 2.2.2. Characterization of Kiwifruit Phenolic Extract

##### Total Phenolic and Flavonoid Content

Total phenolic content (TPC) and total flavonoid content (TFC) were measured using a UV-visible spectrophotometer, model Hitachi U-2000 from Hitachi High Technology (Tokyo, Japan).

For TPC, the Folin–Ciocalteu method was followed [20] with adaptations by Direito et al. (2019) [21]. A volume of 200 µL of Folin–Ciocalteu reagent previously diluted was added to 100 µL of kiwifruit extract. After 3 min, 1 mL of 15% sodium carbonate solution (*w*/*v*) and 2 mL of water were added to the mixture. Measurements were taken after 1 h in the dark at 765 nm. Simultaneously, a blank sample and gallic acid in a standard concentration range of 10–300 mg/L were prepared under the same conditions. All samples and concentration solutions were made in triplicate, and the results were expressed in mg of gallic acid equivalents (GAE) per 100 g of fresh weight (FW).

TFC was determined using the method of Zhishen et al. (1999) [22], with adaptations by Çam & Hişil (2010) [23]. A volume of 1 mL of kiwifruit phenolic extract was added to 4 mL of water and 300 µL of 5% sodium nitrite (*w*/*v*). The solution was left to rest for 5 min. Then, 300 µL of 10% aluminum chloride (*w*/*v*) was added, and after 6 min, 2 mL of 1 M sodium hydroxide was added. Water was added to bring the total volume to 10 mL. Absorbance was measured at 510 nm. In parallel, a blank sample and catechin in a standard concentration range of 20–100 mg/L were prepared. All samples and concentration solutions were made in triplicate, and the results were expressed in mg of catechin equivalents (CE) per 100 g of fresh weight (FW).

#### 2.2.3. Identification of Phenolic Compounds by HPLC-DAD-MS/MS

To identify phenolic compounds, the kiwifruit extract was analyzed by HPLC-DAD-MS/MS, according to Bento-Silva et al. (2020) [24]. Extracts were analyzed on an Alliance 2695 separation module HPLC system (Waters, Dublin, Ireland) coupled to a 2996 Photodiode Array Detector and a Micromass^®^ Quattro Micro triple quadrupole (TQ) (Waters, Dublin, Ireland). The chromatographic separation procedure was carried out using a Lichrocart^®^ RP-18 column (250 × 4 mm, 5 µm) and a Manu-cart^®^ RP-18 precolumn (Merck, Darmstadt, Germany) in a thermostatic oven at 35 °C. The injection volume was 20 µL. The mobile phase consisted of water with 0.5% formic acid as eluent A and acetonitrile as eluent B at a flow rate of 0.30 mL min^−1^. The system was run with a gradient program: 0–15 min from 1 to 10% B, 15–20 min from 10 to 11% B, 20–30 min at 11% B, 30–45 min from 11 to 15% B, 45–55 min at 15% B, 55–95 min from 15 to 30% B, 95–150 min at 30% B, 150–160 min from 30 to 50% B, 160–180 min at 50% B. Returning to the initial conditions took 20 min. The DAD scan was from 210 to 600 nm.

Tandem mass spectrometry (MS/MS) detection was performed using an electrospray ionization source (ESI) operating in positive and negative modes at 120 °C, applying a capillary voltage of 3.0 kV, cone voltage of 30 V, and collision energies of 10, 20, and 30 eV. High-purity nitrogen (N_2_) was used both as the drying gas and nebulizing gas. Ultra-high purity Argon (Ar) was used as the collision gas. To control analytical conditions and collect data, MassLynx software, version 4.1 (Waters, Dubkin, Ireland) was used. For the identification of phenolic compounds, results from the fragmentation pattern of compounds were compared with data published in the literature.

#### 2.2.4. Determination of Antioxidant Activity of the Kiwifruit Phenolic Extract

Antioxidant activity was determined according to the DPPH method [21,25]. Aliquots of 10 µL of extract were mixed with 990 µL of DPPH solution (0.002% in 70% ethanol). The mixture was incubated at room temperature for 30 min in the dark. Two controls were used: a positive control with quercetin at 10 mg/mL in water and an absorbance control with 10 µL of water and 990 µL of DPPH solution. Absorbance was measured at 517 nm against a blank sample of 90% ethanol using a UV-visible spectrophotometer (Hitachi U-2000, Hitachi High Technology, Tokyo, Japan).

The extract was tested in triplicate, and the DPPH sequestration capacity was calculated using the following Formula (1):

(1) $$Scavenging\;activity\left(\%\right)=\frac{Absorbance\;control-Absorbance\;sample}{Absorbance\;control}\times 100$$

#### 2.2.5. Protein Extraction

Kiwifruit was peeled, sliced, and powdered under liquid nitrogen. Polyvinylpolypyrrolidone (0.5 g PVPP per g of fresh weight) and a protease inhibitor cocktail (Complete, EDTA-free inhibitor from Roche (Basel, Switzerland)) were added. Different extractions were performed with three different buffers: (1) Tris-HCl 100 mM, pH 7.5; (2) Tris-HCl 100 mM, pH 8.3 containing 500 mM NaCl [26]; (3) Sodium citrate 100 mM, pH 5.0 [27]. All extractions were carried out for 2 h at 4 °C, followed by centrifugation at 16,863× *g* for 30 min (Beckman Coulter Allegra 25R, Rotor TS-5.1-500 Horizontal, Pasadena, CA, USA). The supernatant was filtered with gauze and stored at –80 °C until further assays were performed.

#### 2.2.6. Polypeptide and Lectin Characterization

The study of polypeptide profile was performed by SDS-PAGE-R (polyacrylamide gel, under denatured and reduced conditions) was performed to characterize the polypeptide profile, according to Laemmli (1970) [28], using 17.5% (*w*/*v*) acrylamide slab gels. Before electrophoresis, all samples were boiled for 3 min in the presence of a sample buffer (80 mM Tris-HCl, pH 6.8, with 2% (*w*/*v*) SDS, 15% (*v*/*v*) glycerol, 0.01% (*w*/*v*) m-cresol purple, and 0.1 M β-mercaptoethanol). Gels were then stained with silver nitrate [29].

A two-dimensional (2D) electrophoresis (IEF/SDS–PAGE-R) of kiwifruit protein extract was carried out according to Oliveira et al. (2019) [15] for proteomic study. Kiwifruit protein extract (1600 µg) was applied in the first dimension (isoelectric focusing (IEF)) and separated using the IPGphor system (Bio-Rad, Hercules, CA, USA). The IPG strips (Bio-Rad, Hercules, CA, USA) were 11 cm in length, with a pH 3–10 gradient.

In the second dimension (SDS-PAGE-R), the gel strips were agitated for 15 min in 50 mM Tris-HCl buffer, pH 8.8, containing 6 M urea, 30% (*v*/*v*) glycerol, 2% (*w*/*v*) SDS, and 1% (*w*/*v*) dithiothreitol. The strips were agitated again with a similar solution but with 2.5% (*w*/*v*) iodoacetamide instead of dithiothreitol. They were placed on top of a 17.5% (*w*/*v*) acrylamide SDS-PAGE gel and sealed with 0.7% (*w*/*v*) agarose. Electrophoresis was performed using a SE 600 Rubi model (Hoefer S1), starting with 220 V, 15 mA for 15 min, followed by 220 V, 30 mA. After electrophoresis was completed, the gels were stained with silver nitrate [29].

The glycosidic nature of the polypeptide constituents of the kiwifruit protein extract was determined after the electrophoretic run (SDS-PAGE-R), followed by transfer to a nitrocellulose membrane using a Trans-Blot SD, Semi-dry Transfer Cell (Bio-Rad, Hercules, CA, USA), for 1 h 15 min at 15 V. Detection was done using the concanavalin A-Peroxidase method, according to Faye & Chrispeels (1985) [30].

#### 2.2.7. Hemagglutination Assays

##### Erythrocyte Cells

Erythrocytes from rabbit blood (Probiológica, Belas, Portugal) were treated according to Ribeiro et al. (2012) [31]. Five mL of blood were washed three times in saline (NaCl 0.9%) and incubated with trypsin from porcine pancreas (Sigma-Aldrich, St. Louis, MO, USA) for 1 h at 37 °C at a final concentration of 1% (*v*/*v*). Finally, 4% (*v*/*v*) suspension of the trypsinized erythrocytes was stored at 4 °C and used for the hemagglutination activity assays.

##### Hemagglutination Activity

For hemagglutination activity quantification [31], protein extracts (100–250 µg in 50–70 µL saline containing 2 mM CaCl_2_ and 2 mM MgCl_2_) were serially diluted (1:2) in a 96-well microplate. The erythrocyte suspension (50–70 µL) was added, and the microplate was incubated for 30 min at 37 °C before visual analysis. Positive (Con-A lectin at 0.5 mg/mL) and negative (saline) controls were prepared. The hemagglutination unit (HU) was defined as the minimum protein concentration required to induce rabbit erythrocyte hemagglutination.

##### Hemagglutination Inhibition

For hemagglutination inhibition by sugar assay [31], 50–70 µL of each sugar solution (0.1 M in saline containing 2 mM CaCl_2_ and 2 mM MgCl_2_, except for L-fucose, which was tested at 0.3 M) was serially diluted (1:2) in a 96-well microplate. A total of 17 sugars were assayed: (1) d-glucose, (2) d-glucosamine, (3) *N*-acetyl-d-glucosamine, (4) d-galactose, (5) d-galactosamine, (6) *N*-acetyl-d-galactosamine, (7) lactose, (8) d-mannose, (9) raffinose, (10) l-fucose (0.3 M), (11) melezitose, (12) α-methyl-d-glucopyranoside, (13) α-methyl-d-mannopyranoside, (14) sucrose; (15) maltose, (16) sialic acid, and (17) l(−)-fucose (0.3 M). After sugar dilution, 4 HU of the kiwifruit protein extract (in 50–70 µL saline containing 2 mM CaCl_2_ and 2 mM MgCl_2_) was added, and the plate was sealed and incubated for 1 h at room temperature. Finally, the erythrocyte suspension at (4% (*v*/*v*) in 50–70 µL saline containing 2 mM CaCl_2_ and 2 mM MgCl_2_) was added, and the microplate was incubated for 1 h at room temperature. The agglutination of the erythrocytes was macroscopically visible and recorded. Appropriate controls were used as described before. Sugar specificity was evaluated by the minimum inhibitory concentration (MIC) of sugar that could inhibit hemagglutination activity.

#### 2.2.8. Purification of Sialic Acid Lectins

Purification of sialic acid lectins was performed using a Fetuin separopore 4B column (BioWorld, Dublin, OH, USA) following BioWorld’s manufacturing protocol. Kiwifruit protein extract was equilibrated with a binding buffer (50 mM sodium phosphate, pH 7.5, containing 100 mM NaCl). The column was balanced at 10 volumes of binding buffer, and the extract was applied, collecting the result. The column was then washed with 10 volumes of binding buffer, and protein elution from the column matrix was performed using elution buffer (10 mM ethanolamine with 150 mM NaCl). Aliquots of 1 mL were collected and monitored at 280 nm (Shimadzu UV-2101PC Spectrometer, Kyoto, Japan). The eluates from each peak were gathered and concentrated by ultrafiltration (Centrifugal Filters Amicon Ultra 15 and 4, Ultra cell 10K, Merck Millipore, Darmstadt, Germany), followed by SDS-PAGE-R to characterize the polypeptides that had bound by affinity to the fetuin column.

#### 2.2.9. Lectin Binding to HT-29 and MIA PaCa-2 Cells

##### Isolation of HT-29 and MIA PaCa-2 Cell Membranes

HT-29 cells were seeded in plastic flasks and cultured Roswell Park Memorial Institute (RPMI) medium, and MIA PaCa-2 cells were cultured in Dulbecco’s modified Eagle’s medium (DMEM), both supplemented with 10% (*v*/*v*) fetal calf serum, 2 mM glutamine, 0.5% (*v*/*v*) of penicillin solution (2 × 104 UI/mL), and 34 mM streptomycin [15]. The cells were kept in a CO_2_ incubator chamber (Sheldon Manufacturing, Cornelius, OR, USA) in a humidified atmosphere containing 5% CO_2_. The medium was refreshed every second day, and the cells were trypsinized when confluence reached 80–100%.

HT-29 and MIA PaCa-2 cells were grown, collected by trypsinization, and stored at –80 °C. Membrane purification was performed according to Vercoutter-Edouart et al. (2008) [32]. The preserved cells were rapidly thawed at 37 °C, then resuspended with 10 volumes of HES buffer (20 mM HEPES, pH 7.4, and 250 mM sucrose), followed by centrifugation at 750× *g* for 10 min at 21 °C. The supernatant was discarded, and the pellet was washed twice with HES buffer containing a protease inhibitor cocktail (without EDTA, Roche (Basel, Switzerland)). Cell lysis was performed by cryolysis, with cells subjected to four cycles of freezing and thawing for 30 min at −20 °C, followed by 20 min of sonication in an ultrasound bath. After centrifugation at 960× *g* for 10 min at 4 °C, the pellet was discarded, and the supernatant was ultracentrifuged at 126,000× *g* for 45 min at 4 °C (Beckman J2-21 m/E, Rotor SW 32 Ti, Pasadena, CA, USA). The resulting pellet, containing the cell membranes, was solubilized in 2 mL of physiological saline (0.9% NaCl) containing 2 mM of CaCl_2_ and 2 mM of MgCl_2_, then divided into aliquots containing 1 mg of protein (determined by the Bradford method [33]) and stored at −80 °C until use.

##### Affinity Binding of Polypeptides vs. Lectins from Total Protein Extract to HT-29 and MIA PaCa-2 Cells Membranes

To evaluate the affinity binding of kiwifruit protein extract to HT-29 and MIA PaCa-2 cell membranes, the protocol by Oliveira et al. (2019) was followed [15]. The isolated cell membranes were individually incubated with kiwifruit protein extract. About 1600 µg of each protein membrane was solubilized in 3 mL of saline containing 2 mM CaCl_2_ and 2 mM MgCl_2_ and incubated for 35 min at 25 °C with gentle agitation, with 1300 µg of kiwifruit protein extract dissolved in 4 mL of saline containing 2 mM CaCl_2_ and 2 mM MgCl_2_. After incubation, preliminary centrifugation was performed (for HT-29 cell membranes at 12,100× *g*; for MIA PaCa-2 cell membranes at 16,500× *g*) to remove the unbound proteins to membranes remaining in the supernatant, followed by two washes of pellet with 15 volumes of saline containing salts and centrifuged under the same conditions for 10 min at 4 °C. The final pellet, containing bound protein and membranes, was solubilized in saline solution (containing salts) and used to evaluate the polypeptides bound to the membranes by SDS-PAGE-R.

#### 2.2.10. Statistical Analysis

The results were processed using Microsoft^®^ Office Excel 2010. Results were expressed as mean ± standard deviation or percentages. The values presented are the mean values of three individual samples (*n* = 3). Statistical analysis was performed using analysis of variance (ANOVA) and Student’s *t*-test.

## 3. Results

### 3.1. Characterization of Phenolic and Flavonoid Compounds

To characterize the phenolic and flavonoid compounds, the first step was to analyze the total phenolic and flavonoid content. The total phenolic content of kiwifruit extract obtained was 193 ± 4.07 mg GAE/100 g FW, and the total flavonoid content was 47.7 ± 0.98 mg CE/100 g FW.

**Figure 1:**
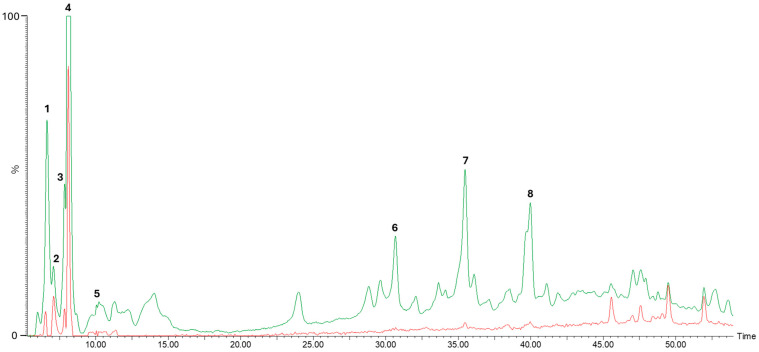
Comparison of chromatographic profiles of kiwifruit extract at 280 nm (green) and 360 nm (red).

To conduct the putative identification of phenolic compounds in kiwifruit extract, a chromatogram was obtained at 280 nm for the detection of all phenolic compounds and others at 360 nm, in order to evaluate the presence of flavonoids (Figure 1).

Table 1 shows the tentatively identified compounds, ordered according to their retention times. The peak numbers described in the chromatogram (Figure 1) correspond to the compounds putatively identified in the following table.

According to Table 1, the main phenolic compounds found in the kiwifruit extract were hydroxycinnamic acids, namely caffeic acid derivatives (caffeoyl glucose and two isomers of caffeic acid hexoside) and 2-hydroxybenzoic acid.

### 3.2. Determination of Antioxidant Activity (DDPH) of Kiwifruit Phenolic Extract

**Figure 2:**
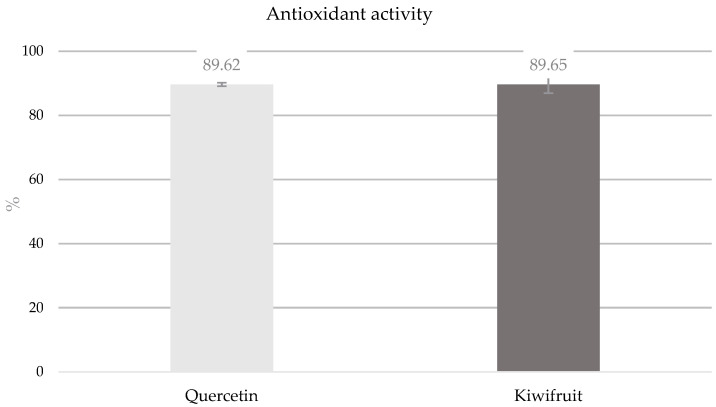
Antioxidant activity of kiwifruit extract and quercetin (positive control) measured by the DPPH method.

Antioxidant activity was measured using the DPPH method, and the results are shown in Figure 2. Quercetin was used as a control to ensure the efficacy of the method. Quercetin and the kiwifruit extract exhibited similar antioxidant activities, indicating that the kiwifruit extract has strong antioxidant potential.

### 3.3. Polypeptide Characterization

#### 3.3.1. Polypeptide Profile

**Figure 3:**
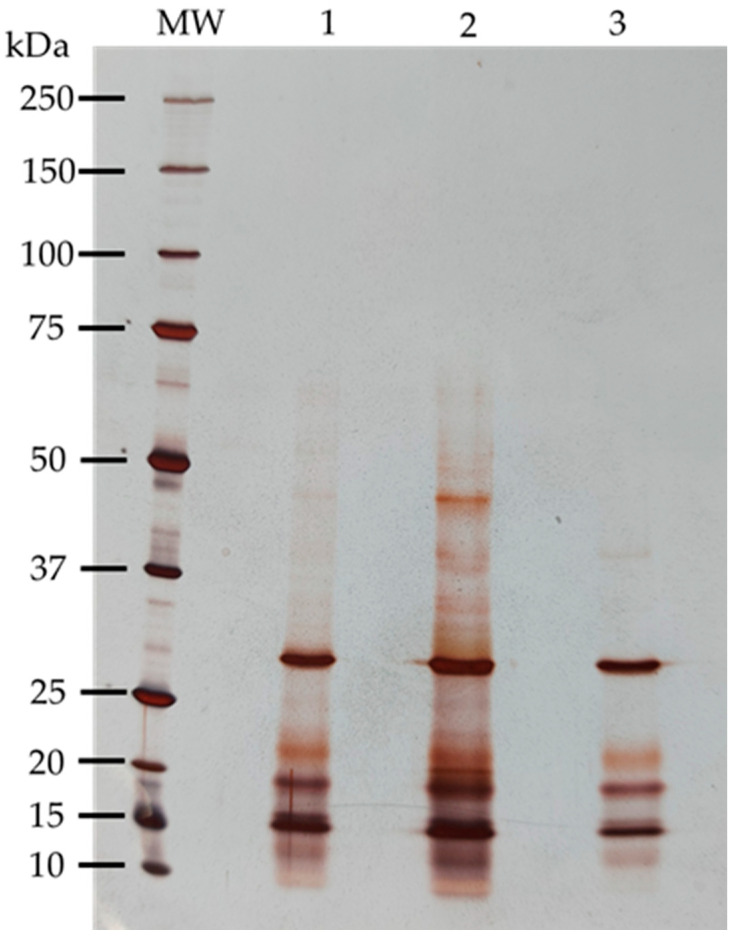
Characterization of kiwifruit protein extract (PE) by different extraction methods. In a 17.5% SDS-PAGE-R (*m*/*v*) acrylamide gel, stained with AgNO_3_, 3 µL of molecular weight marker (MW) was applied, along with 18 µg of total protein extract for methods 1 and 3, and 15 µg for protein extract from method 2.

The proteins from kiwifruit were extracted by three different methods, each with a different buffer, to evaluate the broadest polypeptide coverage. SDS-PAGE-R was performed, as represented in Figure 3, showing the differences between the methods. All methods extracted the most representative polypeptide bands, approximately 15, 20 and 31 kDa. However, method 2 was selected to proceed because it extracted more bands, offering a broader polypeptide profile between approximately 10 to 65 kDa, showing a strong representation of low molecular weight polypeptide bands. Method 1 extracted polypeptides between approximately 10 to 47 kDa, and method 3 was the least comprehensive, extracting only between approximately 12 to 31 kDa.

**Figure 4:**
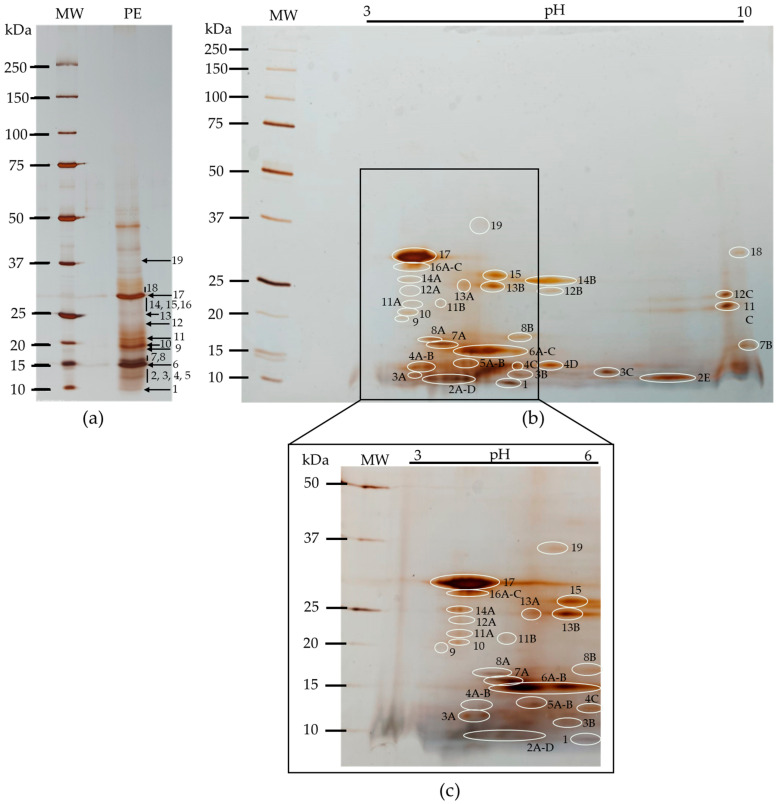
Two-dimensional analysis (IEF/SDS-PAGE-R) of kiwifruit protein extract (PE). (**a**) Characterization of kiwifruit PE in a 17.5% SDS-PAGE-R (*m*/*v*) acrylamide gel, stained with AgNO_3_ as a control. Three µL of molecular weight marker (MW) and 15 µg of PE were applied; (**b**) 2D analysis in a 17.5% IEF/SDS-PAGE-R (*m*/*v*) acrylamide gel, stained with AgNO_3_, pH 3–10. 8 µL of MW and 1600 µg of PE were applied; (**c**) 2D analysis zoom.

The proteomic analysis of the kiwifruit protein extract was evaluated by 2D electrophoresis (IEF/SDS–PAGE-R). The kiwifruit protein extract showed peptides covering the entire pH range (3–10), with a higher concentration in the pH 3–6 range and a low molecular weight area. Peptides with molecular weights above approximately 37 kDa did not appear in proteomic analysis. Some polypeptide bands (Figure 4a) had multiple spots when analyzed in 2D, with bands 2, 6 and 17 being the most representative, as shown in Figure 4b.

Table 2 shows the correspondence between the peptides highlighted in Figure 3 by molecular weight and isoelectric point (pI).

#### 3.3.2. Glycoprotein Detection

**Figure 5:**
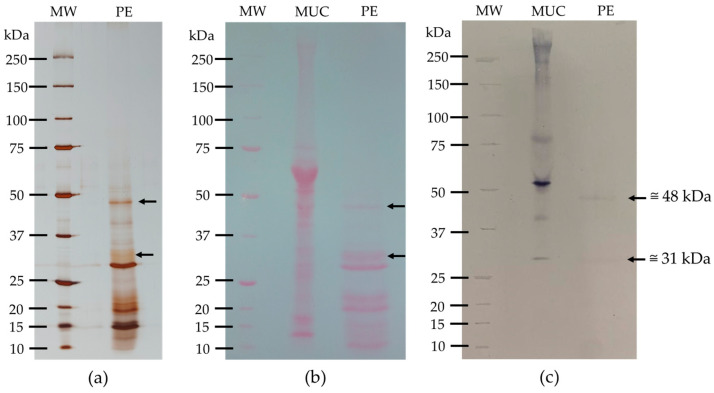
Glycoprotein detection in kiwifruit protein extract (PE). (**a**) Characterization of kiwifruit PE in a 17.5% SDS-PAGE-R (*m*/*v*) acrylamide gel, stained with AgNO_3_ as a control. 3 µL of molecular weight marker (MW) and 15 µg of PE were applied; (**b**) Nitrocellulose membrane transfer stained with Ponceau-S. 3 µL of MW, 50 µg of mucin (MUC), and 200 µg of PE were applied; (**c**) Glycosidic polypeptide detection. 3 µL of MW, 50 µg of mucin (MUC), and 200 µg of PE were applied.

Glycoprotein detection was performed using the concanavalin A-peroxidase method. Two SDS-PAGE-R gels were made: one to control the polypeptide profile (Figure 5a) and the other to control the transfer efficiency to the nitrocellulose membrane (Figure 5b). The glycodetection on the nitrocellulose membrane (Figure 5c) revealed the glycosidic polypeptides present in the protein extract. From the results in Figure 5, it can be deduced that the kiwifruit protein extract contains two glycosylated bands, one of approximately 48 kDa and the other of approximately 31 kDa, indicating that the kiwifruit protein extract is low in glycoproteins.

### 3.4. Lectin Characterization

#### 3.4.1. Hemagglutination Activity and Inhibition

The presence of lectins in the kiwifruit protein extract was realized by hemagglutination assays. Kiwifruit protein extract was prepared and assayed for hemagglutination activity using trypsinized rabbit erythrocytes. Some 150–250 µg of protein was applied to determine the hemagglutination unit (HU).

This assay is fundamental for assessing the activity of lectins and provides important information about the strength of this activity. The lower the HU, the higher the lectin activity. For the kiwifruit protein extract, optimal activity was obtained with an HU of 15.6 µg.

Hemagglutination activity inhibition by sugars was performed after determining the HU. This assay shows the affinity of lectins present in the kiwifruit protein extract for a panel of sugars. These assays were performed with 4 HU of protein for a panel of 17 sugars at a concentration of 0.1 M, except for fucose, which was tested at 0.3 M. The results are presented in Table 3.

Kiwifruit protein extract showed carbohydrate specificity to sialic acid (MIC = 1.56 × 10^−3^ M), d-glucosamine, and d-galactosamine (both with MIC = 0.1 M).

#### 3.4.2. Purification of Sialic Acid Lectins

**Figure 6:**
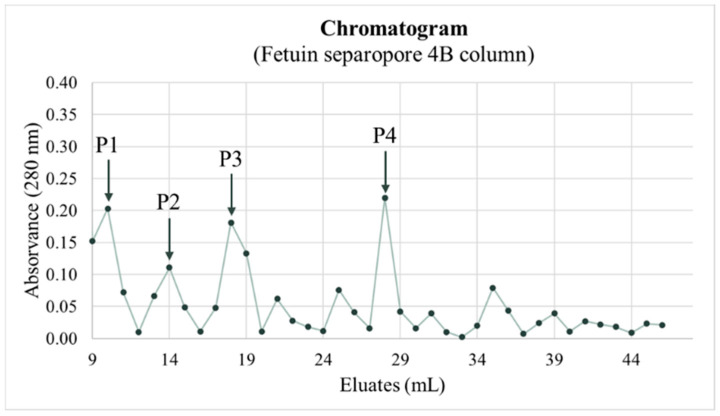
Chromatogram of purification of sialic acid-specific lectins using the Fetuin separopore 4B column. Eluates were collected in 1 mL aliquots and monitored at 280 nm.

The high affinity of kiwifruit protein extract to sialic acid led this team to purify specific lectins for sialic acid using a fetuin column (Fetuin separopore 4B column, BioWorld, Dublin, OH, USA). The chromatogram obtained showed four peaks separated by different elutions (Figure 6).

**Figure 7:**
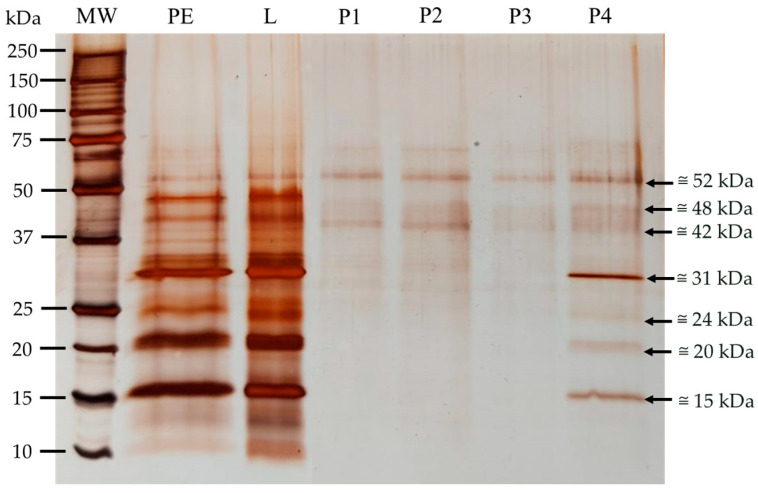
Polypeptide profile of purified sialic acid-specific lectins from kiwifruit protein extract (PE) using the Fetuin separopore 4B column. SDS-PAGE-R, 17.5% (m/v) acrylamide gel, stained with AgNO_3_, shows the profile of the eluates collected. 1.5 µL of molecular weight marker (MW), 8 µg of PE; 8 µg of the loading sample (L), and 30 µL of eluates from each peak (P1–P4) were applied.

The eluates from each peak were collected and concentrated by ultrafiltration (Merck-Amicon system with a 10 kDa cut-off), followed by SDS-PAGE-R to characterize the polypeptides that bound by affinity to the fetuin column (Figure 7). Some polypeptides were purified; however, different peaks showed similar profiles, which can suggest that proteins did not eluate at the same time, despite the fact that the elution buffer always contained 100 mM NaCl.

To validate the sialic acid lectins purification, lectin activity was evaluated for each eluate using a hemagglutination assay. The results are shown in Table 4. All eluates revealed hemagglutination activity.

### 3.5. Lectin Binding to HT-29 and MIA PaCa-2

To evaluate the binding of lectins to HT-29 and MIA PaCa-2 cell membranes after incubation, SDS-PAGE-R was performed, as represented in Figure 7, with the respective controls of cell membranes and the kiwifruit protein extract.

**Figure 8:**
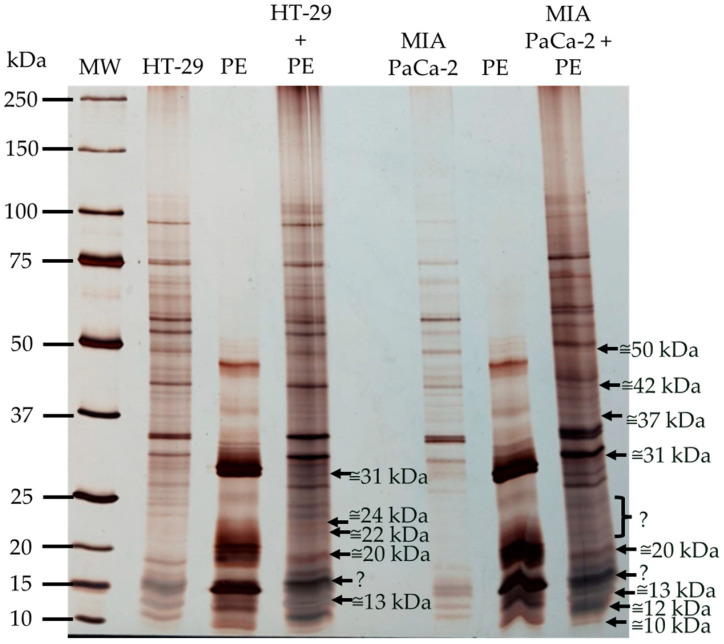
Binding of kiwifruit polypeptides to glycosylated epitopes of HT-29 and MIA PaCa-2 cell membranes. SDS-PAGE-R, 17.5% (*m*/*v*) acrylamide gel, stained with AgNO_3_, shows the profile of the incubation of kiwifruit polypeptides with HT-29 and MIA PaCa-2 cell membranes. 3 µL of molecular weight marker (MW), 15 µg of kiwifruit protein extract (PE), 13 µg of HT-29 control; 13 µg of MIA PaCa-2 control, 15 µg of protein extract incubated with HT-29 (HT-29 + PE), and 15 µg of protein extract incubated with MIA PaCa-2 (MIA PaCa-2 + PE) were applied. The symbol «?» indicates doubts about the existence of polypeptide binding to the membranes.

Both incubations revealed polypeptides bound to the membranes. The HT-29 incubation showed five bound polypeptides, and the MIA PaCa-2 incubation showed eight bound polypeptides. In Figure 8, there are some questionable points due to uncertainty about the existence of polypeptides bound to the membranes. At approximately 15 kDa, the existence of bound polypeptides cannot be confirmed because a very representative polypeptide band was present in both membranes. At approximately 20–25 kDa in the MIA PaCa-2 incubation, the questionable point represents doubt about bound polypeptides due to the poor resolution of the MIA PaCa-2 control.

Table 5 summarizes the results of the various assays for the lectins under study, including glycoprotein detection, binding to HT-29 and MIA PaCa-2 cell membranes, and sialic acid-specific lectin purification.

## 4. Discussion

Kiwifruit consumption has increased over the last few decades, as have studies about the benefits of this fruit [2]. It is important to understand how beneficial this fruit can be to the quality of life of those who consume it, improving knowledge of its bioactivities. Different kinds of studies have been performed, mainly focused on phenolic content and related antioxidant bioactivities, but none has focused on lectin bioactivity. In this study, this team aimed to add new information about the presence of lectins and their bioactivities in kiwifruit.

In this work, the total phenolic content (TPC, mg GAE/100 g FW) was 193 ± 4.07. Other studies show some discrepancies: Liang et al. (2021) reported an acetone-ethanol extract with a TPC of 109 ± 3.03 mg GAE/100 g FW, lower than the present results [39]; Zhang et al. (2020) measured an ethanol extract with 78.04 ± 3.27 mg GAE/100 g FW [40], a very different result from that obtained by Fiorentino et al. (2009) with an ethanol extract, 269 ± 2.07 mg GAE/100 g FW [41]. Total flavonoid content (TFC, mg CE/100 g FW) was 47.7 ± 0.98, an average value compared to other studies: 10.25 ± 2.13 mg CE/100 g FW [40] and 131.7 ± 3.42 mg CE/100 g FW [41]. According to the results obtained and compared with published literature, there are some divergences, but not all authors used the same extraction method. In addition, total phenolic and flavonoid content is affected by many factors such as the varieties used, the type of soil, climatic conditions, time of year, and ripeness of the fruit.

A study compared the TPC of kiwifruit (278 mg GAE/100 g FW) with other fruits. It was noted that kiwifruit has a lower phenolic content than red fruits such as blackberry (660 mg GAE/100 g FW) and strawberry (368 mg GAE/100 g FW). Red fruits are known for their strong antioxidant power, due to their high content of phenolic compounds. However, when compared to traditional fruits, kiwifruit has a higher TPC than various fruits: banana (231 mg GAE/100 g FW), grapes (175 mg GAE/100 g FW), and peaches (163 mg GAE/100 g FW). Kiwifruit has lower phenolic content compared to orange (337 mg GAE/100 g FW) and plum (366 mg GAE/100 g FW) [42].

It was possible to detect several peaks by HPLC-DAD-MS/MS analyses (Figure 1), corresponding to phenolic compounds. Four of them were putatively identified as hydroxycinnamic acids (Table 1), a group of phenolic acids that exhibit various physiological functions, such as antioxidant, anti-inflammatory, and antimicrobial activities [43].

The identified compounds (peaks 1, 2, 3, 4, 5, 7, and 8) had already been described in other articles that analyzed the same variety of kiwifruit (*Actinidia deliciosa* var. Hayward) [5,34,35,36,38].

Phenolic compounds have been widely praised in recent times for their beneficial properties for humans. They are characterized by their high antioxidant power, which provides health benefits. Oxidation is a risk factor for several diseases and also induces inflammation, which is another risk factor in the early development of chronic non-communicable diseases, such as obesity, diabetes, CVD, and cancer, which, as previously mentioned, are considered the main causes of death in recent years [6].

Due to the association between exposure to reactive oxygen species and the development of cancer and cardiovascular diseases, antioxidants have been considered a promising route for the prevention of these diseases. Thus, exogenous antioxidants transmitted through food (through diets rich in fruits and vegetables, for example) become especially important to reinforce endogenous antioxidant mechanisms [44,45].

Because phenolic compounds can act as antioxidants in several ways—through the elimination of reactive species, the ability to capture and trap reactive species, and through metal chelation [46]—they have strong antioxidant properties that help prevent oxidative stress and, as such, to prevent DNA mutations, and, consequently, the appearance of some types of cancer. Additionally, some phenolic compounds have demonstrated the ability to prevent angiogenesis [47]. For this reason, these compounds are present in various nutraceutical, pharmaceutical, medicinal, and cosmetic applications [48,49].

Antioxidant activity obtained by the DPPH method was 89.65% (Figure 2), which is similar to the values published in the literature, 90.37% [50] and 96.09% [51]. However, this team only carried out the DPPH method, and other methods may yield different results. The antioxidant activity in vivo may differ from the DPPH method because it depends on the bioavailability of the antioxidant compounds that are ingested [52,53].

One of our main objectives was to study the composition of lectins in this variety of kiwifruit. By definition, lectins are proteins of non-immune origin, universally distributed in plants, animals, bacteria, and fungi, containing at least one noncatalytic domain, which allows recognition and reversible binding to specific carbohydrates present on glycoproteins and glycolipids, without altering the structure of the sugar. The total protein extract from kiwifruit was essential to evaluate the protein character of lectins. Three different extraction methods were tested to select the best method. The evaluation of the polypeptide profile was performed to identify which of the three methods produced the most complete polypeptide profile. The results in Figure 3 show that each method demonstrated different polypeptide profiles with varying levels of complexity, with method 2 (Tris-HCl 100 mM, pH 8.3 containing 500 mM NaCl) [26] revealing the most complex polypeptide profile, including polypeptides with more extensive molecular weights. This method showed polypeptide bands between approximately 10 and 65 kDa, most of which were of low molecular weights, leading us to select it. In proteomic evaluation, the polypeptide profile of the selected kiwifruit protein extract covered the entire pH range (3–10) (Figure 4), but was more concentrated at pH 3–6 and in the low molecular weight range, as expected, since 1-D polyprotein profile (Figure 3) showed most bands in the lower molecular weight range. The majority of polypeptides have several isoforms spread across the pH range, as shown in Table 2. Glycoprotein detection (Figure 5) revealed that the kiwifruit protein extract contained only two glycoproteins, approximately 31 and 48 kDa, indicating that kiwifruit has a low representation of glycoproteins.

To evaluate lectin characteristics, lectin activity and sugar specificity were determined by hemagglutination activity. The evaluation of lectin activity in the kiwifruit protein extract was performed to determine the presence of lectins and, consequently, their activity. Lectin activity was detected for the first time in the kiwifruit protein extract, with H.U. = 15.6 µg, a promising result since it had never been described before. The hemagglutination inhibition assays were essential to characterize the sugar-binding specificity of the lectins present in the kiwifruit protein extract. Hemagglutination activity was detected for a panel of 17 sugars at a concentration of 0.1 M, except for fucose (0.3 M), using 4 H.U. of protein, as shown in Table 3. The results revealed that only three of the tested sugars had any specificity for the kiwifruit protein extract: *D*-glucosamine, *D*-galactosamine, and sialic acid. *D*-glucosamine and *D*-galactosamine demonstrated poor specificity, with MIC = 0.1 M; however, sialic acid showed a higher specificity for the kiwifruit protein extract, with MIC = 1.56 × 10^−3^ M. The specificity of sialic acid for the kiwifruit protein extract is new and very interesting information, given that sialic acid is essential for various physiological processes in the human body [54].

It is well known that in the tumor process, sialylation is one of the most important glycomic modifications undergone by the tumor cell glycome, forming part of the glycomic aberration, which is a hallmark of cancer. The increased expression of sialic acid, driven by the overexpression of sialyltransferases, is responsible for several effects: tumor growth, metastasis, immune system invasion, and drug resistance [54,55]. Lectins are known to have antitumor activity due to their binding to aberrant glycosylated receptors on tumor cells, which can induce cell death through various mechanisms or even minimize tumor’s irrigation capacity (angiogenesis) [56].

The purification of sialic acid-specific lectins revealed four peaks, as shown in the chromatogram (Figure 6), which were subsequently collected and concentrated for the characterization of the peaks’ polypeptide profiles. The SDS-PAGE-R, presented in Figure 7, showed that the polypeptide profiles of the various peaks are similar, suggesting that the polypeptide profiles did not eluate simultaneously. Peak 4 contains all the sialic acid-specific polypeptides that eluted from the kiwifruit protein extract, with eluates from peak 4 being more evident, with bands at approximately 15, 20, 24, and 31 kDa, and others at approximately 42, 48, and 52 kDa. Since the gel was run under reducing conditions, it appears that 2–3 lectins are involved with similar specificity to sialic acid. To confirm that the purified polypeptides corresponded to lectins, lectin activity was evaluated by hemagglutination assays. Although this team was unable to determine the HU due to the limited protein sample, high lectin activity was observed for peaks 3 and 4, showing the highest specificity, as hemagglutination was observed until the last well of the assay (Table 4), indicating very strong activity.

Since glycomic aberration by sialic acid is an important feature of tumor cells, the team studied the binding capacity of kiwifruit lectins to recognize glycans like sialic acid in tumor cell membranes through their binding. The binding capacity of the lectins present in the kiwifruit protein extract to the membranes of the two tested tumor cell lines (HT-29 and MIA PaCa-2) was evaluated through incubation, followed by characterization (Figure 8). Some polypeptide bands bound to the membranes during incubation, with MIA PaCa-2 showing a higher number of bound polypeptides.

When all the assay results were analyzed (Table 5), it became clear that certain polypeptide bands were consistently present in various experiments, particularly the polypeptide band of approximately 31 kDa, which appeared in all assays. Sialic acid-specific polypeptide bands correspond to some of the polypeptides found in previous assays, except for those at approximately 15 and 52 kDa. The majority of the polypeptide bands were bound to the tumor membranes studied, likely because tumor cell membranes have many glycoproteins with sialic acid residues covering their glycome, making up 40–60% of the cell surface [9]. These sialic acids form a relationship with glycoproteins and lipids through sialyltransferases. The expression of sialyltransferases is positively regulated, leading to the accumulation of sialic acid. The high expression of sialic acid is associated with the promotion of tumor metastasis through various pathways, such as immune evasion and tumor cell survival, which stimulate tumor invasion and migration [54]. Immune evasion leads to the suppression of NK cells, allowing tumor cells to survive [54,57]. 

Some of the polypeptide bands bound to the membranes are not specific to sialic acid; however, the kiwifruit protein extract demonstrated poor specificity for D-glucosamine and D-galactosamine, which may explain the binding of these polypeptides to the glycosylated receptors on the membranes. It could also be related to specificity for another sugar not included in the panel of 17 sugars studied, although these are the most common in glycomic aberration. 

## 5. Conclusions

A diet rich in phenolic compounds can prevent the early development of some chronic diseases, particularly cancer, as well as inhibit cellular growth and metastasis. The Kiwi studied is rich in total phenolic compound with significant antioxidant activity, which suggested that being included in human nutrition could be an asset in preventing the early development of some diseases. Lectins from kiwifruit show great activity for sialic acid, which is uncommon in plant lectins, as sialic acid is a glycan typically expressed in animal bodies. Understanding the importance of sialic acid in the tumor process, we can consider the potential of lectins to investigate their antitumor activity in in vivo models of pancreatic and colorectal cancer as they can binding to both cell lines by sialic acid recognizing.

The bioactivities of kiwifruit explored in this work, due to its phenolic compound composition and the functionalities of lectins, suggest that its consumption can be beneficial for humans, especially due to its role in preventing cancer and metastatic evasion through a cell death mechanism promoted by lectins. Obviously, further studies are required to fully understand the mechanisms involved and the bioavailability of bioactive compounds in humans.

In conclusion, the results suggest that kiwifruit has enormous potential in preventing certain diseases due to the bioactive compounds it contains, and its inclusion in the diet could be important for promoting public health through in vivo evaluation of dose-response. Particularly, the results obtained with the lectins detected, as well as the hypothesis that they could help prevent tumor metastasis, are completely new and suggest that this study has opened another path to combat serious disease. However, it is necessary to continue exploring these mechanisms through in vitro and in vivo assays.

## Figures and Tables

**Table 1:** Putative identification of compounds present in the kiwifruit extract.

No.	RT (min)	Putative Identification	λmax (nm)	Ionization (ESI+/ESI−)	*m/z*	MS2 Product Ions	References
1	6.36	Caffeoyl glucose	269	[M − H]^−^	341	59, 89, 101, 119, **161**, **179**	[34]
2	7.23	2-Hydroxybenzoic acid	274	[M + H]^+^	139	65, 77, **93**, 95	[34]
3	7.95	Malic acid	240	[M − H]^−^	133	**71, 73, 115**	[5,35]
4	8.29	*cis*-Aconitic acid	254	[M − H]^−^	173	67, **111**	[35,36]
5	10.36	Citric acid	253	[M − H]^−^	191	**85, 87, 111**	[5,35]
6	29.84	2-Methylquinoline	283	[M + H]^+^	144	91, **115, 117**, **143, 144**	[37]
7	35.63	Caffeic acid hexoside isomer 1	316	[M − H]^−^	341	71, **135, 179**	[38]
8	40.11	Caffeic acid hexoside isomer 2	283	[M − H]^−^	341	118, **135, 179**, 194, 364	[38]

No. Peak number; RT: retention time. Bold: characteristic fragment ions described by other authors [5,34,35,36,37,38].

**Table 2:** Correspondence between peptides and isoforms obtained by 2D analysis by molecular weight (MW) and isoelectric point (pI).

No.	MW (kDa)	pI
1	10	5.7
2 (A–E)	11	4.3; 4.5; 4.7; 4.9; 8.4–8.9
3 (A–C)	12	3.9; 5.9; 7.4
4 (A–D)	13	3.9; 4.2; 5.8; 6.4
5 (A–B)	14	4.8; 5
6 (A–C)	15	5; 5.4; 5.7
7 (A–B)	16	4.3–4.6; 10
8 (A–B)	17	4.2; 5.9
9	19	3.7
10	20	3.8
11 (A–C)	22	3.9; 4.4; 9.7
12 (A–C)	24	3.8; 6.4; 9.6
13 (A–B)	25	4.8; 5.4
14 (A–B)	26	3.9; 6.2–6.7
15	27	5.4
16 (A–C)	29	3.7; 3.9; 4
17	31	3.6–4.3
18	32	9.9
19	37	5.1

**Table 3:** Inhibition of hemagglutination activity of lectins from kiwifruit total protein extract by sugars.

Sugars		Kiwifruit Protein Extract
No.	(0.1 M)	Sugar Minimal Inhibitory Concentration (MIC) (M)
1	d-Glucose	UD
2	d-Glucosamine	0.1
3	*N*-Acetyl-d-glucosamine	UD
4	d-Galactose	UD
5	d-Galactosamine	0.1
6	*N*-Acetyl-d-galactosamine	UD
7	Lactose	UD
8	d-Mannose	UD
9	Raffinose	UD
10	l-Fucose *	UD
11	Melezitose	UD
12	α-Methyl-d-glucopyranoside	UD
13	α-Methyl-d-mannopyranoside	UD
14	Sucrose	UD
15	Maltose	UD
16	Sialic acid	1.56 × 10^−3^
17	l(−)-Fucose *	UD

UD: undetectable. * 0.3 M of fucose was applied.

**Table 4:** Hemagglutination activity detection in purified eluates of selected peaks by sialic acid affinity column.

	Hemagglutination Activity (H.U.)
Peak 1	Until 1:512 dilution
Peak 2	Until 1:1024 dilution
Peak 3	<1:2048 dilution
Peak 4	<1:2048 dilution

**Table 5:** Match between the polypeptide profile of kiwifruit, polypeptide glycosidic character, and polypeptides specifically binding to HT-29 and MIA PaCa-2 tumor cells.

Molecular Weight (kDa)	2D Spots Analysis	Glycoprotein Detection	Lectin Binding to HT-29 Membranes	Lectin Binding to MIA PaCa-2 Membranes	Sialic Acid Specific Lectin Purification
10	1			+	
12	2			+	
13	3		+	+	
15	4		?	?	+
20	6		+	+	+
22	10		+	?	
24	11		+	?	+
25	12			?	
31	13	+	+	+	+
37	17			+	
42	19			+	+
48	-	+			+
50	-			+	
52	-				+

The symbol «?» indicates doubts about the existence of polypeptide binding to the membranes.

## Data Availability

The original contributions presented in the study are included in the article, further inquiries can be directed to the corresponding author.
